# Hyperprolactinemia with normal serum prolactin: Its clinical significance

**DOI:** 10.4103/0974-1208.69334

**Published:** 2010

**Authors:** Manika Agarwal, Ananya Das, Santa A Singh

**Affiliations:** Department of Obstetrics and Gynecology, North Eastern Indira Gandhi Regional Institute of Health and Medical Sciences (NEIGRIHMS), Mawdiangdiang, Shillong - 793 018, Meghalaya, India

**Keywords:** Amenorrhea, hyperprolactinemia, infertility

## Abstract

Amenorrhea and infertility with an added feature of galactorrhea makes a provisional diagnosis of hyperprolactinemia. But again, normal serum prolactin with all clinical features of hyperprolactinemia might question the diagnosis and further management. The answer lies in the heterogeneity of the peptide hormone - the immunoactive and the bioactive forms. This has been further illustrated with the help of a case which had been treated with cabergoline.

## INTRODUCTION

Amenorrhea with infertility has long been challenging to the treating physician. A high prolactin level is encountered in about one-third of women with no obvious cause of amenorrhea. Again, only one-third of women with high prolactin levels have galactorrhea, probably because the low estrogen environment associated with the amenorrhea prevents a normal response to prolactin.[1]

Galactorrhea or inappropriate lactation is a relatively common problem that occurs in approximately 20-25% of women.[2] Galactorrhea is defined as the presence of a milky nipple discharge at anytime in men and women with no recent history of pregnancy or lactation. In most patho-physiological states, the final common pathway leading to galactorrhea is an inappropriate augmentation of prolactin release. It may be due to pituitary tumors, drugs that inhibit hypothalamic dopamine, hypothyroidism, excessive estrogen intake e.g. oral contraceptives, stress or hypothalamic lesion. Hyperprolactinemia may be associated with a variety of menstrual cycle disturbances: particularly in polycystic ovarian syndrome (PCOS), corpus luteum insufficiency as well as amenorrhea. In patients with both galactorrhea and amenorrhea, approximately two-thirds will have hyperprolactinemia.[3] Pathologic hyperprolactinemia inhibits the pulsatile secretion of gonadotrophin releasing hormone (GnRH) and the reduction of circulatory prolactin level restores menstrual function.

But then the question arises, does galactorrhea with normal serum prolactin need treatment? The answer is yes, if it is associated with any other symptom such as oligomenorrhoea or infertility. This will be further elucidated with the following case history. However, galactorrhea when an incidental finding during examination that has never been noticed by the patient and also without any menstrual irregularity or raised prolactin level may be just kept on a follow-up.

## CASE REPORT

A 31-year-old lady P1L1 with last childbirth eight years back presented to the Gynecology Out Patient Department of our hospital with the complaint of amenorrhea for the last two years. Prior to that, she had normal menstrual cycle. On breast examination, she had secretion from both the breasts, which was not noticed by her earlier. On pelvic examination, uterus, cervix and adnexae were normal. In view of amenorrhea with galactorrhea, she was advised serum prolactin and thyroid profile estimations. Her serum prolactin was found to be 9.96n gm/ml by chemiluminescence assay, which is within normal limits. Her thyroid profile was also normal. Ultrasound showed normal uterus and cervix. She was advised tablet Cabergolin 0.5 mg once a week. She took this treatment for four months but remained amenorrhoeic. She discontinued her treatment, but one month later, she got her menstrual cycle for one day with normal flow. After that, she again came with amenorrhea of two months and her urine pregnancy test was found to be positive. Ultrasound confirmed a seven weeks gestation. Patient had a normal antenatal period and at term delivered a healthy baby vaginally.

## DISCUSSION

Isolated galactorrhea, with normal menstrual cycle and normal serum prolactin levels, has been estimated to occur in up to 20% of women at some point in their lives.[4] Even in our case, the lady had galactorrhea and amenorrhea with normal serum prolactin level. The explanation for such clinically illogical situation can be found in the variable molecular heterogeneity of the peptide hormone. The various forms are associated with varying bioactivity (manifested by galactorrhea) and immunoreactivity (recognition by immunoassay). The predominant variant is little prolactin (80-85%) which also has more biological activity than big prolactin. Macroprolactin or big prolactin is a complex of prolactin with immunoglobulin (IgG) that *in vivo* appears to have limited or no biological activity, possibly because of the failure of the high-molecular weight complex to cross capillary walls.[5] Both the big and big-big forms of hyperprolactinemia have been reported mostly in women with idiopathic hyperprolactinemia and normal menstrual cycle and fertility. It has been suggested that both big prolactin has decreased receptor affinity, whereas big-big prolactin has decreased bioavailability.[6]

Nahid Eftekhan in his study on many infertile women, who had undergone all infertility tests and had galactorrhea, concludes that all gynecologists should consider galactorrhea even in women with normal serum prolactin.[7] Patients with isolated galactorrhea and normal serum prolactin levels do not require treatment if they are not bothered by galactorrhea, do not wish to conceive and do not show evidence of hypogonadism or reduced bone density.[8] The decision to treat galactorrhea should be based on the serum prolactin level, the severity of galactorrhea and the patient’s fertility desires.[9] Hughes *et al*, in his study even concluded the trial of prolactin reducing drugs worthwhile in women with unexplained subfertility and expressible galactorrhea.[10]

## CONCLUSION

Problems in gynecologic endocrinology are as challenging and taxing to the clinician as the problem of amenorrhea. In such situations, the solution of the problem might be found in the heterogeneity of the peptide hormone, where the bioactive form may not be immunoreactive. Sometimes, the solution of big gynecological problems can be solved if we know the small molecule in details.
